# Retinopathy of Prematurity in Very Low Birthweight Neonates of Gestation Less Than 32 weeks in Malaysia

**DOI:** 10.1007/s12098-023-04997-9

**Published:** 2024-01-11

**Authors:** Nem Yun Boo, Ee Lee Ang, Eric Boon-Kuang Ang

**Affiliations:** 1 Department of Population Medicine, Faculty of Medicine and Health Sciences, Universiti Tunku Abdul Rahman, Jalan Sungai Long, Bandar Sungai Long, Kajang, 43000 Selangor Malaysia; 2 Department of Pediatrics, Tengku Ampuan Rahimah Hospital, Ministry of Health of Malaysia, Klang, Selangor Malaysia; 3 Department of Pediatrics, Sultanah Bahiyah Hospital, Ministry of Health, Alor Setar, Kedah Malaysia

**Keywords:** Malaysia, Retinopathy of prematurity, Risk factors, Very preterm, Extremely preterm, Very low birthweight

## Abstract

**Objectives:**

To determine the screening rates and incidence of retinopathy of prematurity (ROP), and risk factors associated with ROP in very low birthweight (VLBW, <1500 g) neonates of gestation <32 wk admitted to neonatal intensive care units (NICUs) in a middle-income country.

**Methods:**

It was a retrospective cohort study of prospectively submitted data by 44 Malaysian NICUs participating in the Malaysian National Neonatal Registry. All VLBW neonates of gestation <32 wk born in 2015–2020 and survived to discharge were included.

**Results:**

Of 11768 survivors, 90.5% (n = 10436) had ROP screening; 16.1% (1685/10436) had ROP. ROP was significantly more common in neonates <28 wk gestation (extremely preterm, EPT) than ≥28 wk gestation (37.7% vs. 9.7%; *p* <0.001), and more common in those with birthweight <1000 g (extremely low birthweight, ELBW) than ≥1000 g (32.9% vs. 9.1%; *p* <0.001). Multiple logistic regression analysis showed that the significant independent factors associated with increased risk of ROP were ELBW, EPT, Indian ethnic group, vaginal delivery, mechanical ventilation >5 d, high frequency ventilation, total parenteral nutrition, late-onset sepsis, bronchopulmonary dysplasia, and intraventricular hemorrhage. Receiving oxygen therapy at birth was associated with significantly lower risk of ROP.

**Conclusions:**

The incidence and severity of ROP increased with decreasing gestation and birthweight. Prolonged duration of oxygen therapy, infection, invasive respiratory support, and conditions commonly causing fluctuations of oxygenation were significant factors associated with increased risk of ROP. Receiving oxygen at birth did not increase risk.

**Supplementary Information:**

The online version contains supplementary material available at 10.1007/s12098-023-04997-9.

## Introduction

Retinopathy of prematurity (ROP) is a condition affecting premature neonates. It results from interruption of normal neurovascular development of the retina which stimulates abnormal compensatory vascularisation following premature birth. The outcome of this neovascularisation varies from complete recovery in mild ROP, to retinal hemorrhage, scarring, detachment, and blindness [1]. ROP-related visual impairment affects the neurodevelopment, learning and quality of life of the child and poses a major social and economic burden to society [2].

In high-income countries (HICs), incidence of ROP ranged from 90% in neonates born at gestation 24 wk to <40% in those born at gestation ≥32 wk [3]. Studies from HICs identified decreasing gestational age [4, 5], decreasing birthweight [4, 5], small-for-gestational age [6], longer duration of ventilation [7] as significant risk factors associated with ROP. In low- and middle-income countries (LMICs), population studies on ROP screening, incidence and risk factors associated with ROP in neonates of gestation <32 wk are limited. To help improve long-term outcome and quality of life of these neonates in resource limited LMICs, such information is extremely important to help guide management policy to reduce the incidence and severity of this condition.

In Malaysia, since 2005 the Ministry of Health has published a clinical practice guideline (CPG) on ROP screening and management [8]. This CPG recommends screening to be “performed at 4–6 wk after birth on all neonates of birthweight <1500 g (very low birthweight, VLBW), gestational age <32 wk, or infants with an unstable clinical course; screening should continue at two- to three-week intervals until the retina is completely vascularised, or ROP has fully regressed with no signs of risk for visual loss, or progressed to a level of severity where treatment is indicated”. The present study aimed to determine the ROP screening rates, its incidence, and risk factors associated with ROP in VLBW neonates of gestation <32 wk, born and admitted to Malaysian neonatal intensive care units (NICUs) in 2015–2020, and survived to discharge.

## Material and Methods

This was a retrospective observational cohort study of neonates admitted to NICUs participating in the Malaysian National Neonatal Registry (MNNR). The MNNR included prospectively collected and anonymised data in a standardised format of all VLBW neonates and those <32 wk gestation. Trained personnel collected these data and merged data of neonates who were admitted more than once. The inclusion criteria for the present study were all VLBW neonates of gestation <32 wk, born in years 2015–2020 and survived to discharge from 44 participating NICUs (which included all major public hospitals, two private hospitals, and one of three university hospitals).

Gestation (in completed weeks) were estimated by antenatal ultrasound findings, maternal last menstrual period, or the New Ballard scores [9] for neonates born to mothers with irregular period and without antenatal ultrasound. Extremely preterm (EPT) neonates were those born at gestation <28 completed weeks, and very preterm (VPT) were those born between 28-<32 completed weeks. Antenatal steroids were steroids given before birth. Outborns were neonates born in non-participating centres. Continuous positive airway pressure therapy (CPAP) commenced shortly after birth was diagnosed as early CPAP (eCPAP). Unwell neonates with positive blood culture at ≤72 h of life were diagnosed to have early-onset sepsis (EOS); those who became symptomatic with positive blood culture after 72 h of life had late-onset sepsis (LOS). Neonates with clinical and radiological features according to Bell’s stage 2 or 3 criteria were diagnosed to have necrotizing enterocolitis (NEC) [10]. Patent ductus arteriosus (PDA) was diagnosed clinically and/or by cardiac ultrasound. Intraventricular hemorrhage (IVH) was diagnosed by cranial ultrasound and severity was graded (0–4) according to Papile’s criteria [11]. Bronchopulmonary dysplasia (BPD) was diagnosed when there was continuous need for oxygen therapy from birth to beyond 36 wk gestation. ROP was diagnosed by ophthalmologists using criteria of the Malaysian CPG [8] adapted from guidelines of the Royal College of Ophthalmologists and the British Association of Perinatal Medicine [12]. Severity of ROP was graded from stages 0 to 5 (with 0 indicating no ROP and 5 indicating highest severity).

The National Medical Ethics and Research Committee of the Ministry Health of Malaysia (NMRR-05-04-168) and the National Institute of Health approved the study and waiver of informed consent (NIH.800-4/4/1 Jld.124 (34)).

The data were analysed using IBM SPSS version 28.0. Categorical variables were summarised as number and percentage, continuous variables as mean±SD, or median and interquartile range (IQR), where appropriate. For between group analysis, Chi square test was used for categorical variables and Student’s T-test or Mann-Whithey U test for continuous variables, where appropriate. Multiple logistic regression analysis was used to identify risk factors significantly associated with ROP. The following potential risk factors were selected a priori for multiple logistic regression analysis: demographic characteristics, history of exposure to oxygen therapy and antenatal steroid, EOS, LOS, and treatment procedures [modes of delivery, resuscitation procedures at birth, surfactant therapy, duration of mechanical ventilation, high frequency ventilation (HFV), total parenteral nutrition (TPN)], and clinical conditions (pneumothorax, NEC, PDA, IVH, and BPD) which were known to cause repeated episodes of oxygen fluctuation in neonates. To check for multicollinearity of independent factors, the variance inflection factors (VIF) were calculated; authors removed factors with VIF >2 before final analysis, and considered *p* values of <0.05 as statistically significant.

## Results

There were 15128 VLBW neonates of gestation <32 wk admitted during this six-year period. Their median gestation was 29.0 wk (IQR: 27.0, 30.0), 31.4% were EPT; median birthweight was 1095 g (IQR: 880, 1290), 37.7% were ELBW; 52.8% were males. There were 63.8% Malay, 9.4% Chinese, 6.0% Indian, 13.9% Malaysians of other ethnicities, and 6.9% foreigners. Of these neonates, 77.8% (n = 11768, Supplementary Fig. S1) survived; none had major congenital malformations.

The average ROP screening rate of survivors was 90.5%, varying from 69.8% to 100% in the 44 NICUs. Six (13.6%) centers screened <80%, 20 (45.5%) centers screened between 80 to <90%, and 18 (40.9%) centers screened ≥90% of their survivors. Compared with those not screened for ROP (n = 1098, 9.5%), neonates who were screened (Table 1) had significantly lower gestation and birthweight, and lower proportions of Malays and foreigners.

**Table 1:** Comparison of demographic characteristics of survivors of very low birthweight neonates of gestation <32 wk with and without ROP screening before discharge from hospitals in MNNR, 2015–2020

**Variables**	**Had ROP screening** **N = 10436**	**No ROP screening** **N = 1098**	***P* values**
Birthweight, g			
Median (IQR)	1130 (955, 1300)	1280 (1100, 1396)	<0.001
<1000 (%)	3095 (29.7)	137 (12.5)	<0.001
1000–1499 (%)	7341 (70.3)	961 (87.5)	
Gestation, weeks			
Median (IQR)	29 (28, 30)	30 (29, 31)	<0.001
<28 (%)	2419 (23.2)	124 (11.3)	<0.001
28-<32	8017 (76.8)	974 (88.7)	
Males (%)	5394/10434 (51.7)	570 (51.9)	0.892
Ethnic groups (%)	N = 10432	N = 1097	
Malay	6647 (63.7)	724 (66.0)	0.005
Chinese	1039 (10.0)	79 (7.2)	
Indian	631 (6.0)	67 (6.1)	
Malaysian of other ethnicity	1467 (14.1)	139 (12.7)	
Foreigner	648 (6.2)	88 (8.0)	

*ROP* Retinopathy of prematurity, *MNNR* Malaysian National Neonatal Registry, *IQR* Interquartile range

**Fig. 1:**
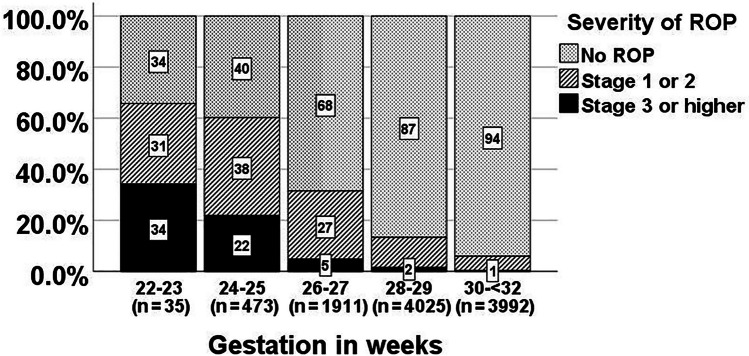
Severity of retinopathy of prematurity (ROP) in different gestational groups

**Fig. 2:**
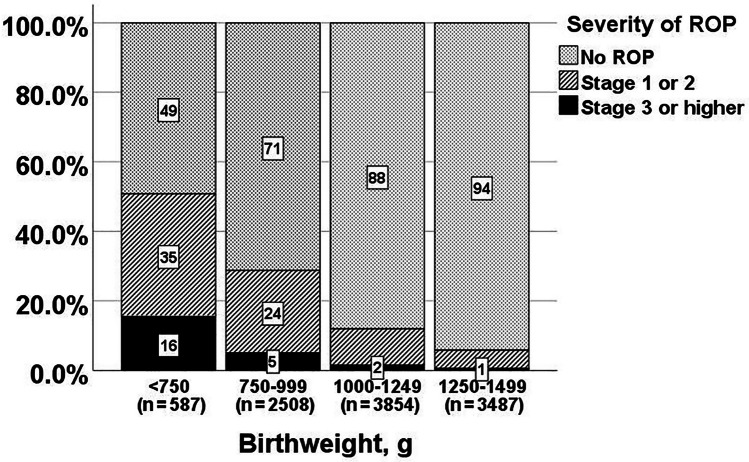
Severity of retinopathy of prematurity (ROP) in different birthweight groups

Of the 10436 neonates screened, 16.1% (n = 1685) had ROP (13.3% mild and 2.9% severe ROP). More than half were stage 1 ROP (56.3%, n = 949); 26.0% (n = 438) were stage 2, 15.0% (n = 253) were stage 3, 1.4% (n = 24) were stage 4, 0.8% (n = 13) were stage 5, and 0.5% (n = 8) had aggressive posterior ROP (APROP); Plus disease was detected in 73 neonates (26 neonates with stage 1 or 2, and 47 neonates with stage 3 or higher). More than half of neonates with ROP were EPT (54.0%, n = 910), and/or extremely low birthweight (<1000 g, ELBW) (60.5%, n = 1019). The incidence and severity of ROP increased with decreasing gestational age (Fig. 1) and deceasing birthweight (Fig. 2). Compared with VPT neonates, a significantly higher proportion of EPT neonates had all stages of ROP (37.7% vs. 9.7%, *p* <0.001) and severe ROP ≥stages 3 (8.6% vs. 1.1%; *p* <0.001). Compared with those weighing ≥1000 g, a significantly higher proportion of ELBW neonates had all stages of ROP (32.9% vs. 9.1%; *p* <0.001) and severe ROP ≥stages 3 (7.0% vs. 1.1%; *p* <0.001). Of the 1685 neonates with ROP, 239 (14.2%) received laser therapy, four (0.2%) received cryotherapy, and 17 (1.0%) had vitrectomy.

When compared with neonates without ROP (Table 2), those with ROP had significantly lower birthweight and gestation, lower proportion of ‘Malaysian of other ethnicity”, higher proportions of outborns and vaginal delivery, lower Apgar scores, lower proportions receiving oxygen and eCPAP at birth; higher proportions receiving bag-and-mask ventilation, endotracheal intubation, chest compression, adrenalin and surfactant therapy at birth; significantly lower admission body temperature; and significantly higher proportions of mechanical ventilation, HFV, TPN, EOS, LOS, pneumothorax, BPD, NEC grades ≥2, PDA and severe IVH. Neonates with ROP had significantly longer median duration of CPAP therapy [15 d (IQR: 5, 30) vs. 6 d (IQR: 2, 15), *p* <0.001], mechanical ventilation [8 d (IQR: 2, 22) vs. 2 d (IQR: 0.5, 6.0), *p* <0.001] and hospitalisation [87 d (IQR: 63, 115) vs. 54 d (IQR: 42, 73), *p* <0.001].

**Table 2:** Comparison of demographic and clinical features between very low birthweight (<1500 g) neonates of gestation <32 wk born in 2015–2020 with and without ROP

**Variables**	**ROP** **N = 1685**	**No ROP** **N = 8751**	***P* values**
Birthweight, g			
Mean (±SD)	954 (218)	1150 (209)	<0.001
<1000	1019 (60.5)	2076 (23.7)	<0.001
1000–1099	666 (39.5)	6675 (76.3)	
Gestation, weeks			
Median (IQR)	27 (26, 29)	29 (28, 30)	<0.001
<28	910 (54.0)	1509 (17.2)	<0.001
28- <32	775 (46.0)	7242 (82.8)	
Intrauterine growth status (%)			
AGA	1432 (85.0)	7595 (86.8)	0.004
SGA	192 (11.4)	956 (10.9)	
LGA	61 (3.6)	200 (2.3)	
Ethnic groups (%)		(N = 8747)	
Malay	1069 (63.4)	5578 (63.8)	0.074
Chinese	190 (11.3)	849 (9.7)	
Indian	133 (7.9)	498 (5.7)	0.162
Malaysian of other ethnicity	186 (11.0)	1281 (14.6)	<0.001
Foreigner	107 (6.4)	541 (6.2)	0.352
Males (%)	855/1684 (50.8)	4539/8750 (51.9)	0.407
Outborns (%)	184 (10.9)	749 (8.6)	0.002
Antenatal steroids (%)	1317/1652 (79.7)	7239/8649 (83.7)	<0.001
Modes of delivery (%)		(N = 8745)	
LSCS	783 (46.5)	5108 (58.4)	
Vacuum or forceps	1 (0.1)	24 (0.3)	0.202
SVD	819 (48.6)	3405 (38.9)	<0.001
Breech	82 (4.9)	208 (2.4)	
Apgar score at 1 min			
Median (IQR)	6 (5, 8)	7 (5, 9)	<0.001
Apgar score at 5 min			
Median (IQR)	8 (7, 9)	9 (8, 9)	<0.001
Treatment at birth (%)			
Oxygen therapy	1236/1638 (75.5)	6949/8595 (80.8)	<0.001
eCPAP	784/1637 (47.9)	5010/8594 (58.3)	<0.001
Bag-and-mask ventilation	1152/1636 (70.4)	5228/8588 (60.9)	<0.001
Endotracheal intubation	1242/1634 (76.0)	5234/8590 (60.9)	<0.001
Chest compression	56/1633 (3.4)	169/8589 (2.0)	<0.001
Resuscitated with adrenalin	29/1633 (1.8)	87/8586 (1.0)	0.008
Surfactant therapy (%)	1439 (85.4)	6347 (72.5)	<0.001
Admission temperature, ^o^C, mean (± SD)	35.7 (1.1)	35.8 (0.9)	<0.001
CPAP in NICU (%)	1591 (94.4)	8223 (94.0)	0.470
CPAP duration (%)		(N = 8750)	
0–14 d	831 (49.3)	6414 (73.3)	
>14 d	854 (50.7)	2336 (26.7)	<0.001
Mechanical ventilation (%)	1516 (90.0)	6736 (77.0)	<0.001
MV duration (%)	(N = 1684)	(N = 8749)	
0–5 d	712 (42.3)	6475 (74.0)	
>5 d	972 (57.7)	2274 (26.0)	<0.001
High frequency ventilation (%)	475 (28.2)	1104 (12.6)	<0.001
Total parenteral nutrition (%)	1527 (90.6)	6909 (79.0)	<0.001
Early-onset sepsis (%)	44 (2.6)	117 (1.3)	<0.001
Late-onset sepsis (%)	284 (16.9)	597 (6.8)	<0.001
Pneumothorax (%)	44 (2.6)	126 (1.4)	<0.001
Bronchopulmonary dysplasia (%)	808 (33.1)	877 (11.0)	<0.001
NEC grade 2 or 3 (%)	130 (7.7)	421 (4.8)	<0.001
Patent ductus arteriosus (%)	997 (59.2)	3325 (38.0)	<0.001
Severity of IVH (%)	(N = 1577)	(N = 7713)	
No IVH	608 (38.6)	4365 (56.6)	<0.001
Grade 1 or 2	753 (47.7)	2930 (38.0)	
Grade 3 or 4	216 (13.7)	418 (5.4)	

*AGA* Appropriate-for-gestational age, *eCPAP* Early continuous positive airway pressure, *IVH* Intraventricular hemorrhage, *IQR* Interquartile range, *LGA* Large-for-gestational age, *LSCS* Lower segment cesarean section, *MV* Mechanical ventilation, *NEC* Necrotizing enterocolitis, *NICU* Neonatal intensive care unit, *ROP* Retinopathy of prematurity, *SD* Standard deviation, *SGA* Small-for-gestational age, *SVD* Spontaneous vertex delivery

Of the 10436 neonates, 8986 (86.1%) had complete set of data of variables needed for multiple logistic regression analysis (no ROP, n = 7483, with ROP, n = 1503). After controlling for various potential confounders (Table 3), the significant independent factors associated with increased risk of ROP were ELBW, EPT, Indian ethnic group, vaginal delivery [Spontaneous vertex delivery (SVD) or breech], mechanical ventilation >5 d, HFV, TPN, LOS, BPD and IVH. Receiving oxygen therapy at birth and “Malaysian of other ethnicity” were associated with significantly lower risk. The *p* value of the Hosmer and Lemeshow test for this model was: 0.332; the *p* value of Omnibus test of model coefficient was <0.001. The area under receiver operation curve (ROC) of the regression equation is: 0.779 (95% CI: 0.765, 0.792; *p* <0.001).

**Table 3:** Multiple logistic regression analysis of potential risk factors associated with ROP in neonates born in 2015–2020

**Variables**	**Adjusted Odds Ratios (95% CI)**	***P* values**
Birthweight, g		
1000–1099	1	
<1000	2.170 (1.853, 2.542)	<0.001
Gestation, weeks		
28-<32	1	
<28	2.368 (2.014, 2.785)	<0.001
Intrauterine growth		
AGA	1	
SGA	1.062 (0.854, 1.319)	0.590
LGA	0.983 (0.699, 1.384)	0.923
Ethnic groups		
Chinese	1	
Malay	1.047 (0.857, 1.281)	0.651
Indian	1.463 (1.089, 1.966)	0.012
Malaysian of other ethnicity	0.770 (0.595, 0.997)	0.047
Foreigner	1.098 (0.806, 1.496)	0.552
Males	0.899 (0.793, 1.019)	0.095
Outborns	1.112 (0.869, 1.424)	0.399
Antenatal steroids	0.891 (0.753, 1.054)	0.178
Modes of delivery		
LSCS	1	
Vacuum or forceps	0.257 (0.031, 2.154)	0.210
Vaginal route	1.324 (1.156, 1.515)	<0.001
Treatment at birth		
Oxygen therapy	0.678 (0.584, 0.788)	<0.001
eCPAP	1.066 (0.933, 1.217)	0.348
BMV	0.973 (0.827, 1.144)	0.741
ETT intubation	1.198 (0.999, 1.437)	0.051
Chest compression	1.048 (0.732, 1.499)	0.799
Surfactant therapy	1.016 (0.837, 1.233)	0.872
Admission temperature, ^o^C	0.967 (0.908, 1.029)	0.285
MV duration		
0–5 d	1	
>5 d	1.428 (1.233, 1.653)	<0.001
HFV	1.202 (1.028, 1.407)	<0.021
TPN	1.259 (1.015, 1.56)	0.036
EOS	1.219 (0.801, 1.854)	0.355
LOS (%)	1.504 (1.252, 1.807)	<0.001
Pneumothorax (%)	0.851 (0.556, 1.302)	0.457
BPD (%)	1.665 (1.440, 1.925)	<0.001
NEC grade 2 or 3 (%)	1.173 (0.922, 1.492)	0.194
PDA (%)	1.143 (1.000, 1.307)	0.050
Severity of IVH		
No IVH	1	
Grade 1 or 2	1.196 (1.046, 1.367)	0.009
Grade 3 or 4	1.541 (1.235, 1.921)	<0.001

*AGA* Appropriate-for-gestational age, *BMV* Bag-and-mask ventilation, *BPD* Bronchopulmonary dysplasia, *CI* Confidence intervals, *EOS* Early-onset sepsis, *ETT* Endotracheal, *eCPAP* Early continuous positive airway pressure, *HFV* High frequency ventilation, *IVH* Intraventricular haemorrhage, *LGA* Large-for-gestational age, *LOS* Late-onset sepsis, *LSCS* Lower segment cesarean section, *MV* Mechanical ventilation, *NEC* Necrotizing enterocolitis, *PDA* Patent ductus arteriosus, *ROP* Retinopathy of prematurity, *SGA* Small-for-gestational age, *TPN* Total parenteral nutrition

## Discussion

In this large Malaysian multicentre study of VLBW neonates of gestation <32 wk, 90.5% of survivors were screened, 16.1% had ROP, and 2.9% had severe ROP. Screening rates varied widely among NICUs. The bigger and more mature neonates had higher rate of missed screening, like study elsewhere [13].

The incidences of all stage ROP and severe ROP in Malaysian NICUs were lower than those reported in Turkey (16.1% vs. 42.0% and 2.1% vs. 11.1%) [14], Sweden (16.1% vs. 41.1%, 2.1% vs. 20.3%) [15], and Taiwan (16,1% vs. 42.5%) [16]. However, severe ROP were much more common in EPT and ELBW neonates in Malaysian NICUs than those in Australian-New Zealand network (37.7% vs. 9.6%) [4].

Besides neonatal demographic characteristics, and duration of oxygen therapy, the role of sepsis, various treatment procedures, and clinical conditions commonly causing fluctuation of oxygenation as potential risk factors associated with ROP were investigated in the present study. This is because both prolonged duration of oxygen therapy and conditions causing fluctuation of oxygenation were found to be significant risk factors in animal studies [17–19]. These factors were found to promote initial compromised retinal physiologic vascularity and delayed development during the first few weeks of life, and subsequent vaso-proliferation. In the present study, the results of multiple regression analysis confirmed these findings.

Unlike study elsewhere [20], PDA was only of borderline significance. Like studies elsewhere, decreasing gestational age, decreasing birthweight [2–4], vaginal delivery [21], TPN [16], LOS [22], IVH [23], and BPD [17] were significant independent factors associated with increased risk of ROP in present cohort. Greater fluctuation of oxygenation could be a possible mechanism for increasing risk of ROP during vaginal delivery than during LSCS.

The present study is the first to report on the effect of oxygen therapy during resuscitation at birth on ROP, and found this brief exposure was associated with significantly lower risk. The risk of ROP was significantly higher only in those receiving prolonged oxygen therapy ≥4 wk in neonates with BPD, as reported previously [16]. Unlike a large Korean neonatal network study [24], which reported mechanical ventilation longer than 2 wk was significant risk factor associated with severe ROP, the present study found ROP risk increased significantly even with shorter duration of >5 d.

Although univariate analysis in this study showed neonates with ROP had higher rates of exposure to endotracheal intubation at birth, multiple logistic regression analysis did not show statistical significance. One possible explanation was shorter duration of fluctuation of oxygenation associated with endotracheal intubation did not pose significant risk.

Both animal and clinical studies reported sepsis-associated inflammation possibly played an important role in ROP [25]. In the present study only LOS, but not EOS, was a significant independent factor associated with increased risk. One possible explanation for this discrepancy could be the small number of neonates with EOS in the present cohort which was under-powered to detect its role on ROP.

Contrary to reports elsewhere [16], SGA was not a significant independent risk factor associated with ROP in the present cohort. The authors found Indian Malaysians had significantly higher risk, and Malaysians of other ethnicity had significantly lower risk of ROP than Chinese and Malay Malaysians. These differences could be due to genetic variants, as extremely preterm neonates with variants in the intronic region of the brain-derived neurotrophic factor (BDNF) had significantly higher risk associated with ROP and severe ROP [26, 27].

Studies elsewhere reported VLBW neonates of <32 wk gestation given fish-oil-rich lipids in TPN had significantly lower risk of ROP than those given soy-based lipid in TPN [28, 29]. In the present study, TPN was a significant independent factor associated with increased risk of ROP. As the MNNR database did not capture information on types of lipids used in TPN, the authors were unsure whether failure to use fish-oil based TPN was an explanation for TPN being a factor associated with increased risk of ROP in Malaysian neonates.

The following are the strengths of this study: it is a large sample-sized multicentre national study from a LMIC; a standardised format was used to collect the data prospectively; and >90% of VLBW/<32-wk-gestation livebirths in Malaysia were admitted to the 44 participating centres during this period.

The main limitations of this study were its inability to report on the roles of other potential risk associated with ROP (including total duration of oxygen therapy, neonatal anemia [30], blood transfusion), reasons for no screening in some neonates, and information on the zone status of ROP in the affected eyes. The MNNR did not include these variables in its database.

The principal new findings in this study are short duration of oxygen exposure at birth is associated with reduced risk, while prolonged duration of exposure to oxygen, invasive respiratory support and fluctuation of oxygenation are associated with increased risk of ROP.

Based on the findings of this study, the ROP screening rates of EPT and ELBW neonates in Malaysian NICUs need further improvement as these neonates have the highest incidence and risk of severe ROP. Avoidance of vaginal delivery, use of oxygen therapy for resuscitation at birth, reduction of duration of mechanical ventilation and HFV, use of fish-oil-based lipids in TPN, and prevention of LOS, IVH and BPD are strategies which may reduce the incidence and severity of ROP in these high-risk neonates.

## Supplementary Information

Below is the link to the electronic supplementary material.

Supplementary Fig. S1 STROBE diagram of patient recruitment. *MNNR* Malaysian National Neonatal Registry, *NICU* Neonatal intensive care unit, *ROP* Retinopathy of prematurity (DOCX 261 KB)

## Data Availability

The data that support the findings of this study are available from the Malaysian National Neonatal Registry, but restrictions are applied to the availability of these data which were used under permission for the current study.
