# Supplementary material for: Retinopathy of Prematurity in Very Low Birthweight Neonates of Gestation Less Than 32 weeks in Malaysia
Source: Indian J Pediatr. 2024 Jan 11;92(3):260–7. doi: 10.1007/s12098-023-04997-9 (PMC11845399; doi:10.1007/s12098-023-04997-9)
Supplement: Supplementary file 1 — Supplementary Fig. S1 STROBE diagram of patient recruitment. MNNR Malaysian National Neonatal Registry, NICU Neonatal intensive care unit, ROP Retinopathy of prematurity (DOCX 261 KB) [file 12098_2023_4997_MOESM1_ESM.docx]

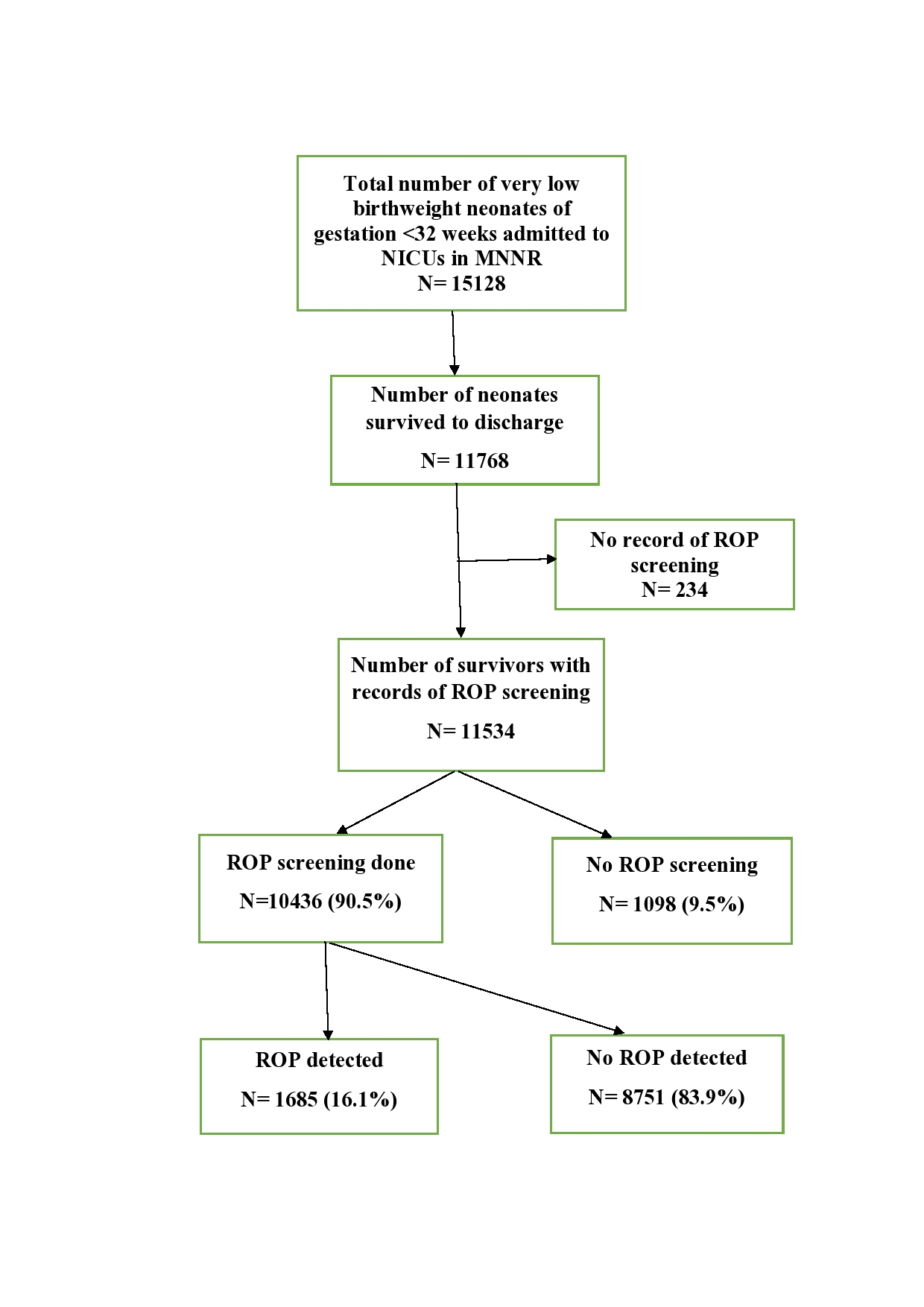


**Supplementary Fig. S1** STROBE diagram of patient recruitment. *MNNR* Malaysian National Neonatal Registry, *NICU* Neonatal intensive care unit, *ROP* Retinopathy of prematurity
